# Peak inflammation in atherosclerosis, primary biliary cirrhosis and autoimmune arthritis is counter-intuitively associated with regulatory T cell enrichment

**DOI:** 10.1016/j.imbio.2015.02.006

**Published:** 2015-08

**Authors:** Stefano Garetto, Anna Elisa Trovato, Ana Lleo, Federica Sala, Elisa Martini, Alexander G. Betz, Giuseppe D. Norata, Pietro Invernizzi, Marinos Kallikourdis

**Affiliations:** a Adaptive Immunity Laboratory, Humanitas Clinical and Research Center, Via Manzoni 56, Rozzano (Milano), Italy; b Liver Unit and Center for Autoimmune Liver Diseases, Humanitas Clinical and Research Center, Via Manzoni 56, Rozzano (Milano), Italy; c Dipartimento di Biotecnologie Mediche e Medicina Traslazionale, Università degli Studi di Milano, Via Manzoni 56, Rozzano (Milan), Italy; d Department of Pharmacological and Biomolecular Sciences, Università degli Studi di Milano, Milan, Italy; e Medical Research Council Laboratory of Molecular Biology, Francis Crick Avenue, Cambridge, UK; f Center for the Study of Atherosclerosis, Società Italiana Studio Aterosclerosi, Ospedale Bassini, Cinisello Balsamo, Italy; g The Blizard Institute, Centre for Diabetes, Barts and The London School of Medicine & Dentistry, Queen Mary University, London, UK; h Division of Rheumatology, Allergy and Clinical Immunology, University of California, Davis, CA, USA

**Keywords:** Regulatory T cells, Atherosclerosis, Primary biliary cirrhosis, Rheumatoid arthritis, Inflammation, Autoimmunity

## Abstract

Regulatory T cells (Treg) influence the development of autoimmunity and their use is increasingly proposed for clinical applications. The well-characterized suppressive potential of Treg frequently leads to the assumption that Treg presence in prevailing numbers is indicative of immunosuppression. We hypothesized that this assumption may be false. We examined models of three different diseases caused by organ-specific autoimmune responses: primary biliary cirrhosis, atherosclerosis and rheumatoid arthritis (RA). We examined indicators of relative abundance of Treg compared to pro-inflammatory T cells, during peak inflammation. In all cases, the results were compatible with a relative enrichment of Treg at the site of inflammation or its most proximal draining lymph node. Conversely, in healthy mice or mice successfully protected from disease *via* a Treg-mediated mechanism, the data did not suggest that any Treg accumulation was occurring. This counter-intuitive finding may appear to be at odds with the immunosuppressive nature of Treg. Yet extensive previous studies in RA show that an accumulation of Treg occurs at peak inflammation, albeit without resulting in suppression, as the Treg suppressive function is overcome by the cytokine-rich environment. We suggest that this is a ubiquitous feature of autoimmune inflammation. Treg abundance in patient samples is increasingly used as an indicator of a state of immunosuppression. We conclude that this strategy should be revisited as it may potentially be a source of misinterpretation.

## Introduction

Foxp3^+^ Regulatory T cells (Treg) affect disease development in autoimmunity and unwanted inflammation (Wing and Sakaguchi, 2010). A large number of studies (Allan et al., 2008), mostly on animal models, have associated defects in Treg cells with the development of autoimmune symptoms (Zhang et al., 2009), whilst experimental administration of Treg protects from autoimmunity (Munoz-Suano et al., 2012). Their use is increasingly proposed for clinical applications (Wing and Sakaguchi, 2010). Whilst aspects of their dynamic behavior during physiological conditions are understood (Liston and Gray, 2014), little is known about how Treg behave during an ongoing autoimmune inflammatory response (Torgerson, 2006; Germain, 2012). Scientists often conceptualize a balance of anti-inflammatory Treg versus pro-inflammatory effector T (Teff) cells, with the most numerous population determining the outcome of the response: tolerance versus autoimmunity. This is increasingly used in clinical studies, examining Treg abundance in patients’ peripheral blood (Kofler et al., 2011). The balance metaphor is likely to be valid during the start of a response, when Treg failure in suppressing Teff may be the initiating event of autoimmunity. We hypothesized that this may not be so during the peak of autoimmunity. In other words, once an autoimmune inflammatory response has been established, Treg may be unable to control it, even if they accumulate in higher numbers in their attempt to do so. Intriguingly, extensive studies in rheumatoid arthritis (RA) have shown that Treg are enriched in the inflamed synovium. Mechanistic insight derived from these studies demonstrates that Treg do not succeed in suppressing the response as they are impeded or overwhelmed by local pro-inflammatory cytokines (Benito-Miguel et al., 2009; Nguyen et al., 2007; Nie et al., 2013). Similar data have also been produced and published for many other autoimmune diseases, even though in most cases the counter-intuitive Treg accumulation was evidenced but not highlighted (Maganto-Garcia et al., 2011; Patel et al., 2010). In some of the older studies, especially from human patients, the difficulty in obtaining patient samples other than peripheral blood, the unreliable identification of Treg cells prior to the availability of Foxp3 as a marker and the more promiscuous expression of Foxp3 as a Treg marker in the human have often inhibited the drawing of firm conclusions (Torgerson, 2006). Yet, especially in the mouse, where Foxp3 expression has a better correlation – albeit not perfect (Miyao et al., 2012; Sharma et al., 2010) – with suppressive function (Rubtsov et al., 2010; Hori, 2011) the data is indeed convincing that such an enrichment takes place (Nguyen et al., 2007). To assess whether this counter-intuitive observation applies to a wider range of diseases, we retrospectively examined unpublished data generated in our laboratories using three different model autoimmune pathologies. Our results, together with the extensive data published in the context of previous studies on different autoimmune diseases, suggest that it may be unwise to use Treg enrichment in an autoimmune inflammation context as an indicator of successful immunosuppression.

## Materials and methods

### Disease models

All animal handling was performed by expert technicians, according to the national legislation and local committee regulations of the authors’ institutes.

### PBC induction

We used the PBC mouse model as described in (Wakabayashi et al., 2008). Female C57BL/6 mice at 6 weeks of age (Charles River) were immunized intra-peritoneally with a mixture of 138-BSA (2-octynoic acid (OA)-BSA; 100 μg/50 μL) in Complete Freund's Adjuvant (CFA; Sigma–Aldrich) containing 10 mg/mL of *Mycobacterium tuberculosis* strain H37Ra and subsequently boosted every 2 weeks, for a total of 8 weeks, with 2OA-BSA and Incomplete Freund's Adjuvant (IFA; Sigma–Aldrich). Pertussis toxin was administered at day 0 and 2 (100 ng/mouse). 2OA (Sigma–Aldrich) was conjugated to BSA as described previously (Wakabayashi et al., 2008). Serum titers of antiPDC-E2 autoantibodies were measured by enzyme-linked immunosorbent assay using standardized recombinant auto antigens.

### Flow cytometry

Single cell suspensions were prepared from harvested tissue by passing through 70 μm cell strainers (BD) and lysing red blood cells using Lysing Buffer (BD). Livers had been additionally previously segmented by scalpel and incubated for 1.5 h in RPMI 1640 medium (Lonza) containing 0.05% collagenase type IV (Sigma–Aldrich) at 37 °C. Treg and Teff populations in harvested lymphoid tissue were assessed by flow cytometry on a FACSCanto II (BD Biosciences) using rat anti-mouse CD4-PerCP-Cy5.5 (BD, clone RM 4-5) and rat anti-mouse FoxP3-AlexaFluor488 (eBioscience, clone FJK-16s) antibodies.

### Atherosclerosis models

Low Density Lipoprotein (LDL) receptor deficient mice or apolipoprotein E (ApoE) deficient mice or C57BL/6 controls were fed ad libitum with an atherogenic western type diet (21% fat, 0.15% cholesterol and 19.5% casein, Harlan) starting at 8 weeks of age, for 16 weeks. Mice were sacrificed for analysis after anesthetization with an overdose of Avertin 2.5% (Aldrich Chemical Co), followed by cervical dislocation. Aorta isolation, mRNA extraction and cDNA preparation were performed as described previously (Norata et al., 2012).

### CIA induction

Induction was performed as described previously (Munoz-Suano et al., 2012). Briefly, female C57BL/6 mice were immunized intradermally with 100 μg chicken collagen II (Sigma–Aldrich) in CFA on day 0 and 21. Mice were monitored for signs of arthritis and for anti-collagen antibodies. The humane end-point was set at a clinical score of ≥8 out of 12. Mice were considered arthritic when having a score ≥3. Joints were obtained during week 6 post-CIA induction, when the incidence reaches a plateau, for mRNA extraction and qPCR analysis. Treg-mediated, pregnancy-associated protection from CIA symptoms, as described previously (Munoz-Suano et al., 2012), was achieved by mating the female mice with allogeneic BALB/c males on day 31 post-CIA induction. RNA was harvested using RNeasy kit (Qiagen). cDNA was synthesized using Superscript II RT (Invitrogen). mRNA expression levels were measured using Taqman Gene Expression assays (Applied Biosystems) for CD3e and custom-made primers for Foxp3 and HPRT, as described previously (Kallikourdis and Betz, 2007).

### Statistical analysis

Statistical analysis was performed with GraphPad Prism software, using unpaired *t*-test for Gaussian or Mann–Whitney test for non-Gaussian distributions, after normality testing.

## Results

**Fig. 1:**
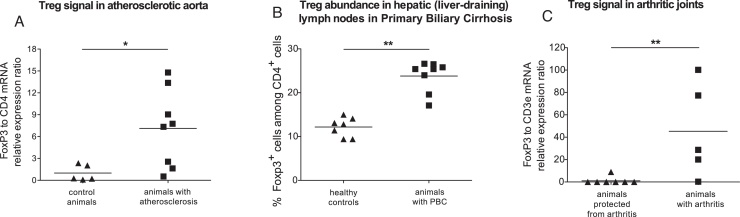
Regulatory T cell enrichment during peak inflammation in mouse models of organ-specific autoimmunity. (A) Atherosclerosis: The ratio of Treg to Teff, as indicated by the relative levels of FoxP3 to CD4 mRNA measured by real-time qPCR analysis, in aortic samples from C57BL/6 control mice or atherosclerotic mice; *P* = 0.0289, unpaired *t* test. Values shown are normalized to the mean of control animals. Each dot represents one animal: *n* = 5 control mice; *n* = 4 ApoE^−/−^ and *n* = 4 LDLR^−/−^ mice, pooled for this analysis (B) Primary biliary cirrhosis: Flow cytometric analysis of FoxP3 positive cells among CD4 positive cells in mice with induced PBC or control C57BL/6 mice. Hepatic lymph nodes; *P* = 0.0014, Mann–Whitney test. Each dot represents one animal: *n* = 7 and 8 animals, pooled from two independent PBC induction preparations. (C) Collagen Induced Arthritis. The ratio of Treg to total T cells, as indicated by the relative levels of FoxP3 to CD3ɛ mRNA measured by real-time qPCR analysis, in the joints of arthritic mice or mice that were protected from CIA-induced arthritis by becoming pregnant; *P* = 0.0028, Mann–Whitney test. Values shown are normalized to the mean of animals protected from arthritis. Each dot represents one animal: *n* = 7 and 5 animals, pooled from two independent CIA induction preparations.

Atherosclerosis is characterized by T cell responses to vascular self-antigens. Mice deficient in the receptor for low-density lipoprotein (LDL) or in apolipoprotein E develop hypercholesterolemia and subsequent atherosclerosis when fed an atherogenic diet (Ammirati et al., 2012). Using real-time qPCR, we examined the ratio of Foxp3 to CD4 mRNA, as an indicator of the relative abundance of Treg in the tissue. The ratio was significantly increased in the ascendant aortic arch of mice with atherosclerosis compared to controls (Fig. 1A; *P* < 0.05, unpaired *t* test).

Primary biliary cirrhosis (PBC) is an autoimmune disease targeting the liver small bile ducts. We used the murine model of PBC, at 8 weeks post-induction, when the inflammation reaches a plateau (Wakabayashi et al., 2008). The percentage of Foxp3^+^ cells within the CD4^+^ T cell population, examined by flow cytometry, was not significantly different in para-aortic lymph nodes and spleens of mice with induced PBC compared to healthy mice (Supplementary Fig. 1A and B). Yet, in the liver-draining hepatic lymph nodes of mice with PBC, CD4^+^ Foxp3^+^ cells were 23.8 ± 1.2% of CD4^+^ T cells, compared to only 12.2 ± 0.8% in healthy controls (Fig. 1B; *P* = 0.0014, non-parametric *t* test). The liver itself in animals with PBC showed a similar significant Treg enrichment (Supplementary Fig. 1C).

Supplementary Fig. S1 related to this article can be found, in the online version, at http://dx.doi.org/10.1016/j.imbio.2015.02.006.

Supplementary Fig. S1 Regulatory T cell enrichment in a mouse model of primary biliary cirrhosis. Flow cytometric analysis of FoxP3 positive cells among CD4 positive cells in mice with induced PBC or control C57BL/6 mice. (A) Para-aortic lymph nodes; *P* > 0.05, unpaired *t* test. (B) Spleen; *P* > 0.05, unpaired *t* test. (C) Liver, P = 0.0061, Mann-Whitney test. As in PBC the tissue affected is restricted to the portal area of the liver, the analysis of whole liver cell suspensions represents only an approximate sampling of the actual target organ of autoimmunity. Each dot represents one animal, pooled from two independent PBC induction preparations.

Pregnancy, *via* the action of Treg, alleviates the symptoms of arthritis, as we have recently shown in the collagen-induced arthritis (CIA) model of RA (Munoz-Suano et al., 2012). We examined, *via* real-time qPCR, joints of mice in which CIA had been induced and compared them with mice in which CIA had been induced but the clinical symptoms were alleviated by pregnancy. We found that arthritic mice had significantly higher (almost 50-fold) ratio of Foxp3 mRNA to T cell-marker CD3e mRNA, in their joints, when compared to mice protected from arthritic symptoms (Fig. 1C; *P* = 0.0028, non-parametric *t* test). This matches previously reported findings in the K/BxN mouse model of rheumatoid arthritis, showing a very high enrichment of Treg in the synovial fluid of arthritic joints, compared to lymph nodes from the same animals (Nguyen et al., 2007). In our experiments, non-arthritic non-pregnant controls had no Foxp3 mRNA signal in their joints, as expected by the lack of any inflammation (data not shown).

## Discussion

In all models examined, during peak inflammation, our findings are compatible with an enrichment of Treg either at the site of disease or its most proximal draining lymph nodes. In atherosclerosis and arthritis these may be tertiary lymphoid structures existing within the affected tissue itself, adjacent to the atherosclerotic plaque (Weih et al., 2012) or in the arthritic synovium (Humby et al., 2009). An important caveat for the interpretation of our arthritis and atherosclerosis data is that Foxp3 has been shown to be expressed in epithelial cells of breast, lung, prostate and intestinal tissue (Chen et al., 2008). Thus we cannot exclude an epithelial source of the Foxp3 signal detected in our assays. However, a large number of studies have demonstrated that once RA is fully established, an enrichment of Treg can be seen in the synovium of human patients, compared to the levels found in peripheral blood (van Amelsfort et al., 2004; Mottonen et al., 2005; Benito-Miguel et al., 2009). This enrichment without resultant immunosuppression would appear to contradict the well-characterized Treg potential to suppress immune responses. Yet the evidence from the RA studies suggests that this enrichment is an insufficient effort of the immune system to limit the ongoing response. The Treg appear to be impaired in their ability to suppress (Notley and Ehrenstein, 2010; Ruprecht et al., 2005) or the pro-inflammatory Teff appear to become refractive to suppression (Benito-Miguel et al., 2009). This impediment has been attributed to the production of pro-inflammatory cytokines such as IL-15, IL-7 or IL-6 (Notley and Ehrenstein, 2010; Benito-Miguel et al., 2009; Ruprecht et al., 2005) by the inflamed synovium. Additionally, TNFα has been shown to lead to FOXP3 dephosphorylation and consequent inhibition of Treg suppressive activity in rheumatoid arthritis patients (Nie et al., 2013). Further, soluble cytokine glucocorticoid-induced TNFR-related protein (sGITR-L) has been shown to abrogate the ability of Treg to suppress *in vitro* (Ji et al., 2004). Collectively, these studies offer clear explanations for the mechanism through which the pro-inflammatory milieu at the site of autoimmune inflammation is paradoxically characterized by lack of suppression despite an accumulation of Treg.

In animal models of RA, therapeutic interventions that lead to a reduction of symptoms are associated with a further enrichment of Treg in the arthritic joint (Morgan et al., 2005; Ko et al., 2010). This lends further support to the notion that the physiological enrichment of immunosuppressive Treg at peak inflammation may represent an unsuccessful or insufficient effort of the immune system to block or limit the ongoing autoimmune response. Indeed, an elegant study in the K/BxN model of RA has shown that Treg are enriched in the synovium of arthritic mice, more so than in secondary lymph nodes; albeit their immunosuppressive effect appears to be overwhelmed by the ongoing inflammation (Nguyen et al., 2007).

A number of studies have examined the relative levels of Treg cells in PBC patients. Yet again, an increase in the number of Treg cells in the affected tissue, in this case the portal ducts of the liver, can be observed in PBC patients compared to healthy controls (Lan et al., 2006; Sasaki et al., 2007; Sakaki et al., 2008; Wang et al., 2010). Our novel results in the mouse model of PBC are consistent with these findings on the Treg enrichment in PBC patients.

Studies in animal models of atherosclerosis have shown that Treg cells exert suppression on the pro-atherosclerotic, pro-inflammatory Teff responses (Ait-Oufella et al., 2006). Therapeutic interventions in models of the disease were associated with expanded Treg numbers in the aorta but not the lung mucosa (Klingenberg et al., 2010). The data we present here is in agreement with two recent studies in the aortas of LDL receptor deficient mouse model of atherosclerosis and in unstable carotid plaques of human patients, which both identified a Treg enrichment (Maganto-Garcia et al., 2011; Patel et al., 2010).

A relative enrichment of Treg at the site of inflammation, at peak of disease, has also been reported in experimental autoimmune encephalomyelitis (EAE), a model of Multiple Sclerosis (MS). As in other diseases, the Treg enrichment was insufficient to control the disease. This was attributed to production of the cytokines IL-6 and TNF at the site of inflammation (Korn et al., 2007); similar findings have also been reported for sarcoidosis (Torgerson, 2006).

Our data, as well the findings reviewed above, suggest a general pattern in the dynamics of Treg cells during the peak phase of autoimmune inflammation: a counter-intuitive, localized enrichment of Treg occurring at the site of inflammation. Treg have the potential to protect from autoimmunity, but once a response has commenced, their natural enrichment at the draining lymphoid structure closest to the target organ is likely to be an insufficient attempt of the immune system to control the inflammation. Conversely, healthy or cured tissue is not enriched in Treg cells.

Yet, somewhat surprisingly, the corollary that Treg levels *per se* make a poor indicator of a state of immunosuppression is rarely highlighted. As a consequence, Treg abundance in patient peripheral blood is increasingly used in clinical and pre-clinical studies as a marker assumed to be proportional to successful suppression (Lan et al., 2006; Kofler et al., 2011). Our findings suggest that the abundance of Treg cells *per se* may not necessarily be an indication of whether the response is being successfully suppressed, as abundance of Treg in draining lymphoid structures may be reflective of more, not less severe autoimmune inflammation. Hence peripheral blood Treg measurement is of limited diagnostic value, and it may potentially become a source of misinterpretation.

## Conflict of interest

The authors declare that they have no competing financial interests.
