# Supplementary figures and images for: Peak inflammation in atherosclerosis, primary biliary cirrhosis and autoimmune arthritis is counter-intuitively associated with regulatory T cell enrichment
Source: Immunobiology. 2015 Aug;220(8):1025–9. doi: 10.1016/j.imbio.2015.02.006 (PMC4457006; doi:10.1016/j.imbio.2015.02.006)

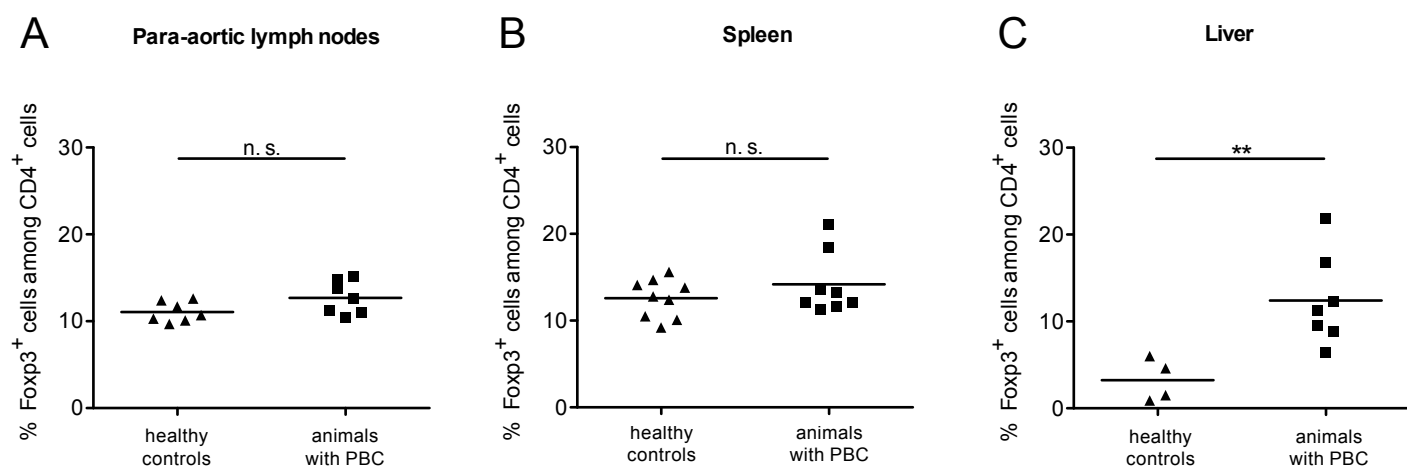

**Supplementary Figure 1**

Supplement: Supplementary Fig. S1 — Regulatory T cell enrichment in a mouse model of primary biliary cirrhosis. Flow cytometric analysis of FoxP3 positive cells among CD4 positive cells in mice with induced PBC or control C57BL/6 mice. (A) Para-aortic lymph nodes; P > 0.05, unpaired t test. (B) Spleen; P > 0.05, unpaired t test. (C) Liver, P = 0.0061, Mann-Whitney test. As in PBC the tissue affected is restricted to the portal area of the liver, the analysis of whole liver cell suspensions represents only an approximate sampling of the actual target organ of autoimmunity. Each dot represents one animal, pooled from two independent PBC induction preparations. [file mmc1.pdf]
